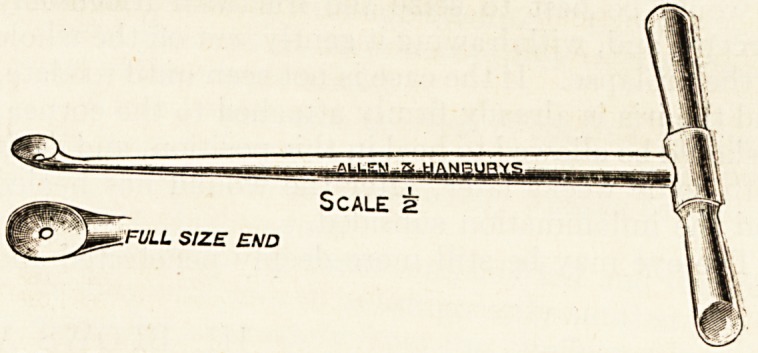# Concerning Operations for Otorrhœa

**Published:** 1907-11-30

**Authors:** 


### Otology.

### CONCERNING OPERATIONS FOR OTORRHŒA.

In a certain proportion of cases of suppuration in the middle ear an abscess forms, intra-cranially, between the bone and the dura mater, just as an extra-cranial abscess may form superficially over the mastoid process. This intra-cranial abscess formation may take place over the roof of the middle ear, beneath the temporo-spheroidal lobe, or in the cerebellar fossa, between the posterior surface of the petrous and the anterior surface of the cerebellum, either deep to the lateral sinus, or in the groove of the sinus, forming a peri-sinous abscess.

In operating upon an extra-dural abscess it is of the highest importance to remember that extra-dural suppuration is frequently accompanied by thrombosis of the lateral sinus or abscess of the brain. In ten successive cases of middle-ear disease with intra-cranial complications, recently observed by the writer, an extra-dural abscess was present in eight. An extra-dural abscess without intra-dural infection existed in only three of these cases, while only two cases of brain abscess occurred without extra-dural suppuration.

Though the figures are too small to possess any considerable statistical value, they emphasise the facts (1) that brain abscess or sinus thrombosis is commonly associated with extra-dural abscess; (2) that an extra-dural abscess may form before the brain or lateral sinus or meninges become affected; (3) that brain abscess, etc., may exist without extra-dural abscess.

The symptoms are rarely sufficiently characteristic to establish a final diagnosis; they are sometimes latent, the existence of the abscess not even being suspected, until the performance of the radical mastoid operation for otorrhœa. The symptom-group of extra-dural abscess, complicating middle-ear suppuration, is rather suggestive than absolute. It is made up of mental irritability, pyrexia, and usually intense pain over the temporal bone area, or diffuse headache. These symptoms are, however, common to other intra-cranial lesions. A rigor may occur and may be repeated, although repeated rigors point more frequently to sinus thrombosis than to extra-dural abscess or to any other single intra-cranial complication.

At best the symptoms are often difficult to differentiate from those due to infection of the lateral sinus, from lepto-meningitis, and, sometimes, from brain abscess. Insomnia, giddiness, vomiting, optic neuritis and nystagmus have all been observed. A large abscess between the bone and the dura, gives rise to symptoms of compression of the brain or local convulsions; spastic paralysis of the opposite side, of the face and arm especially, deepening unconsciousness, and aphasia have, according to some observers, been occasionally met with. Thus the symptoms may resemble those of meningitis, of brain abscess, of septic toxæmia, or of acute localised inflammation of the temporal bone alone.

Pyrexia is the rule. It may be only to 99° or 100°, but there are exacerbations of the fever, the temperature rising to 102° or 104°. The pulse-frequency is increased, to say 100 or more per minute, except in the less common cases of extra-dural compression of the brain, when the pulse is relatively slow, say 60 to 80.

The pain is constant, localised, or radiating, and is nearly always very severe. It possesses the character of bone-pain in its great intensity at night, causing insomnia; there is tenderness to pressure on the area of bone affected, above or behind the meatus, over the base of the mastoid, or over the cerebellar fossa.

In cases of chronic otorrhœa, the onset of any threatening symptoms, such as have been enumerated, often demands immediate operation.

When an extra-dural abscess is suspected, the radical mastoid operation is first performed as described in a previous issue of The Hospital. The mastoid antrum having been opened through a post-aural incision, the posterior and middle cranial fossæ are exposed; the pus rapidly wells up into the wound in a stream which generally pulsates synchronously with the brain. More bone is removed to permit free exit to the pus and enable the operator to obtain full inspection of the dura. To do this the gouge is supplemented with bone forceps, such as Jansen's, or with a cross-handled curette, as illustrated in the text. The dura lining the abscess will be found covered with granulations. Instead of being tough and resistant, the granulating dura may be soft and friable or in a sloughing condition. Any fistulous track towards the brain must be sought for and opened up if present. If the granulations are vascular, bleeding readily, with no slough, if, furthermore, the sinus itself can be identified, is found to be compressible, and refills readily when pressure, gently applied, is removed, we may postpone incision of the sinus, especially so, if no pyæmic symptoms are present.

The radical mastoid operation is completed by meatal drainage, as already described in a previous issue. In those cases where otitis media is recent or acute, the tympanic chamber and ossicles are left intact, as in Schwartz's method, and the post-aural wound is drained by tube. The dressings are at first renewed daily, and the wound heals by granulation; the drainage tube is replaced by smaller tubes, and drainage continued for about two weeks.

In all cases of extra-dural suppuration the subsequent course of the patient after the operation must be watched with especial care. In the event of suspicious symptoms we must be prepared to open the wound and explore for a residual abscess, sinus thrombosis, or brain abscess, without delay.

## Figures and Tables

**Figure f1:**